# Rural adolescent attitudes and use of helmets while riding ATVs, motorcycles and dirt bikes

**DOI:** 10.1186/s40621-024-00532-2

**Published:** 2024-09-05

**Authors:** Charles A. Jennissen, Sehansa R. Karunatilaka, Brianna J. Iverson, Devin E. Spolsdoff, Kristel M. Wetjen, Brenda Vergara, Shannon R. Landers, Pam J. Hoogerwerf

**Affiliations:** 1 Department of Emergency Medicine, Roy J. and Lucille A. Carver College of Medicine, University of Iowa, 200 Hawkins Dr, Iowa City, IA 52242 USA; 2 Stead Family Department of Pediatrics, Roy J. and Lucille A. Carver College of Medicine, University of Iowa, Iowa City, IA USA; 3 College of Liberal Arts and Sciences, University of Iowa, Iowa City, IA USA; 4 Roy J. and Lucille A. Carver College of Medicine, University of Iowa, Iowa City, IA USA; 5 Division of Pediatric Surgery, Department of Surgery, University of Iowa Health Care, Iowa City, USA; 6 Injury Prevention and Community Outreach Program, University of Iowa Health Care Stead Family Children’s Hospital, University of Iowa, Iowa City, IA USA; 7 College of Osteopathic Medicine, Kansas City University, Kansas City, MO USA

**Keywords:** Adolescent, All-terrain vehicle, Dirt bike, Farm, Helmet, Head injury, Law, Motorcycle, Rural, Safety, Youth

## Abstract

**Background:**

Head injuries are the most common cause of death in some motorized vehicles for which helmet use can significantly decrease the risk. Our objective was to determine rural adolescents’ attitudes regarding helmets and their use while riding ATVs, motorcycles and dirt bikes.

**Methods:**

A convenience sample of 2022 Iowa FFA (formerly Future Farmers of America) Leadership Conference attendees were surveyed. After compilation, data were imported into the statistical program, R (https://www.R-project.org/). Descriptive statistics, contingency table, logistic regression and non-parametric alternatives to ANOVA analyses were performed.

**Results:**

1331 adolescents (13–18 years) participated. One half lived on a farm, 21% lived in the country/not on a farm and 28% were from towns. Nearly two-thirds (65%) owned an ATV with 77% of all having ridden one in the past year. Farm residents had the highest ATV ownership (78%) and having ridden (80%) proportions, both *p* < 0.001. Overall, ownership and ridership for motorcycles (22% and 30%, respectively) and dirt bikes (29% and 39%, respectively) was significantly less than ATVs, all *p* < 0.001. Of ATV riders, those living on farms or in the country/not on a farm rode them more frequently than those from towns, *p* < 0.001. Higher percentages always/mostly wore helmets when riding dirt bikes (51%) and motorcycles (57%) relative to ATVs (21%), *p* < 0.001. Those from farms had lower proportions wearing helmets versus those living elsewhere for all vehicles. Helmet use importance ratings (1–10, 10 high) were not different for motorcycles (mean 8.6, median 10) and dirt bikes (mean 8.3, median 10), but much lower for ATVs (mean 6.1, median 6). Females, non-owners, and helmet law supporters all had higher helmet use importance ratings. Males, those from farms, and owners and riders of the vehicles all had lower proportions that supported helmet laws. Support for helmet laws was significantly lower for ATVs (30.7%) than dirt bikes (56.3%) or motorcycles (72.3%), both *p* < 0.001. Those whose families had strict ATV “No Helmet, No Riding” rules had much higher helmet use and helmet importance ratings.

**Conclusions:**

Our study indicates that the safety culture surrounding helmet use is relatively poor among rural adolescents, especially on farms, and deserves targeted interventions.

## Background

All-terrain vehicles (ATVs), dirt bikes and motorcycles are all significant threats for morbidity and mortality in pediatric populations. These motorized vehicles pose particular risks to riders because of their speed and acceleration capabilities, their relative instability, their lack of external protection and seat belts, and their propensity for riders to be thrown in a crash. Data from the National Electronic Injury Surveillance System reveal ATV-related incidents are responsible for an estimated 95,000 emergency department (ED) visits per year with over a third (37%) of those injured being < 18 years old (Zhang 2022; Wiener et al. 2022). In fact, more children under 16 years of age in the U.S. die in ATV-related incidents than from bicycle crashes (Helmkamp et al. 2009). In addition, an estimated 23,800 youth ≤ 19 years are treated in EDs with dirt bike-related injuries per year with 7.5% of these requiring hospitalization (Centers for Disease Control and Prevention (CDC) 2006). Moreover, more than 6,000 U.S. motorcyclists died in 2021 (Institute and for Highway Safety ((IIHS), Highway Loss Data Institute (HLDI). 2023), and motorcyclists have 6 times the fatality rate of passenger car occupants involved in crashes (Blincoe et al. 2010). While the total number of motor vehicle fatalities has decreased, motorcycle crash deaths are now nearly 3 times greater than in 1997 (Institute and for Highway Safety ((IIHS), Highway Loss Data Institute (HLDI). 2023; Blincoe et al. 2010).

Crashes in these three motorized vehicles often result in traumatic head injuries (Larson and McIntosh 2012; Denning et al. 2013a, 2013b, 2014; Denning and Jennissen 2018; Ganga et al. 2023). Adolescents in ATV crashes are more likely to be primarily ejected or in collisions that lead to being thrown or falling from the vehicle than other ATV riders (Denning et al. 2014; Unni et al. 2012). For those injured on ATVs, 16–44% have injuries to the head, neck and face region (Bhutta et al. 2004; Mangano et al. 2006; Collins et al. 2007; Kirkpatrick et al. 2007; Shults et al. 2013). In one study, nearly half of the dirt bike motocross riders suffered at least one episode of concussive symptoms over a 4 month racing season, for which three-quarters required medical treatment (McIntosh and Christophersen 2018). Youth with head trauma, including intracranial hemorrhage, brain contusions and concussions, more often require hospitalization and rehabilitation treatment than children with no neurologic injuries (Bhutta et al. 2004; Mangano et al. 2006; Humphries et al. 2006; Nabaweesi et al. 2018). Moreover, head injuries are the most frequent cause of death in crashes involving ATVs, dirt bikes and motorcycles (Denning et al. 2013a, 2013b, 2014; Bhutta et al. 2004; Mangano et al. 2006; Collins et al. 2007; Kirkpatrick et al. 2007; Shults et al. 2013; Humphries et al. 2006; Barron et al. 2021; Testerman et al. 2018; Helmkamp et al. 2008; Denning and Jennissen 2016; Keenan and Bratton 2004; Miller et al. 2006; Shannon et al. 2018; Bowman and Aitken 2010; Linnaus et al. 2017; Kelleher et al. 2005; GAO 2010).

The use of helmets has been highly effective in preventing head injuries. In fact, for both ATVs and motorcycles, helmets may reduce fatal head injuries by ~ 40% and nonfatal brain injuries by 60% or more (Denning et al. 2013a, 2013b; Bowman et al. 2009; Merrigan et al. 2011; Rodgers 1990; Liu et al. 2008; Coben et al. 2007). Youth wearing helmets on ATVs had lower Injury Severity Scores and shorter hospital stays as compared to those unhelmeted (Gittelman et al. 2006; Holt et al. 2022; Brown et al. 2002). Likewise, ATV crash victims without a head injury had fewer hospital and intensive care unit (ICU) admissions and lower healthcare costs (Bowman et al. 2009; Merrigan et al. 2011; Brown et al. 2002; Carr et al. 2004; Myers et al. 2009). Motorcycle and dirt bike studies have found similar findings (Abdelgawad et al. 2013). Helmeted motorcycle crash victims have fewer injuries to the head and face, less hospital and ICU admissions, less need for mechanical ventilation, and lower mortality (Barron et al. 2021; Khor et al. 2017; Patel et al. 2019; Lawrence et al. 2009). The National Highway Traffic Safety Administration (NHTSA) estimates that for every 100 motorcycle riders killed in crashes without helmets, 37 could have been saved if all had worn a helmet (NHSTA 2021).

Despite the proven benefits of helmets, use among ATV crash victims is generally low (Levy et al. 2023; Denning and Jennissen 2018; Denning et al. 2014; Linnaus et al. 2017; GAO 2010; Holt et al. 2022; Ho et al. 2017). In several survey studies of youth, only 17–45% reported always or almost always wearing a helmet with the most frequent riders often reporting the lowest helmet use (Shults and West 2015; Hafner et al. 2010; Burgus et al. 2009; Jennissen et al. 2014). Dirt bike riders often have higher helmet use than those on ATVs suggesting a stronger safety culture regarding head protection (Nichols et al. 2022; Vittetoe et al. 2022). The National Occupant Protection Use Survey (NOPUS) conducted by NHTSA found that 67% of motorcycle riders wore a Department of Transportation (DOT)-approved helmet in 2022 (Boyle 2023).

Rural adolescents and those living on ranches and farms are a particularly at-risk ATV riding population (Gerberich et al. 2001; Goldcamp et al. 2006; Hendricks et al. 2001; Jennissen et al. 2022). Few published studies have investigated rural teenager’s use of and attitudes towards helmet use on ATVs, and there is even less data regarding their operation of dirt bikes and motorcycles. The objective of our study was to determine rural adolescents’ attitudes regarding helmets while riding ATVs, dirt bikes and motorcycles, the frequency of helmet use on these vehicles, their level of support regarding helmet laws, and how demographic factors may be associated.

## Methods

Survey was conducted among a convenience sample of attendees at the 2022 Iowa FFA Leadership Conference (April 10–12, 2022). FFA, previously known as Future Farmers of America, is a national youth organization that focuses on agricultural education and leadership development. In 2023, there were 19,200 members across 260 Iowa FFA chapters (Iowa FFA Association 2024). Conference participants were recruited at the University of Iowa Stead Family Children’s Hospital (SFCH) safety booth to complete the survey either on paper or electronically via their cell phone onto a software platform (Qualtrics International, Inc, Provo, UT). Surveys were anonymous and completed independently. Written surveys were reviewed for completeness by the safety booth staff. As an incentive for completing the survey, participants were given the opportunity to play a Plinko game to obtain a small prize.

### Survey

Members of the SFCH’s Off-Road Vehicle Task Force developed the survey through a collaborative and iterative process. To ensure validity, surveys were administered to twelve volunteers aged 13–20 years. Participants were encouraged to seek clarification on any unclear sections of the survey. A comparison of both written and verbal responses was conducted to ensure consistency, and final survey design was shaped by these findings.

Demographic variables included age (years), gender (male, female, non-binary, other), where they live (on a farm, in the country but not on a farm, in town) and race/ethnicity (White, Black/African American, Asian, Latino/Latinx, other). The survey included similar questions for sections on ATVs, dirt bikes and motorcycles. Photos of each vehicle were placed next to the name to assure that participants understood the vehicle to which they were being referred.

Survey questions included whether their family owned the vehicle, and the respondent’s frequency of riding the vehicle in the past year with response options: daily, weekly, monthly, just a few times a year or less, and haven’t ridden in the past year. Additionally, participants were queried about their helmet use when on the vehicle in the past year with the following responses: always, most of the time, sometimes, rarely, never, and haven’t ridden in the past year. The importance of helmet-wearing was measured on a scale of 1–10, with 1 being "not important at all" and 10 being "extremely important." For ATVs only, respondents were asked whether their parents had a strict “No helmet, No Riding” rule for them. Finally, participants expressed their opinion on whether they thought there should be a law requiring helmet use when riding each vehicle.

### Data analysis

Electronic and written surveys were provided to the research team for analysis. All conference attendees were allowed to take the survey, but analysis was restricted to participants 13–18 years of age. Since the analysis was being done on an existing, anonymous dataset, the university’s institutional review board determined the study was exempt. Written surveys were entered into Qualtrics with the survey responses previously entered by cell phone. Data were aggregated and exported into the statistical software program, R (https://www.R-project.org/), for analysis.

Descriptive statistics (frequencies), contingency table (chi-square, Fisher’s exact test), and multivariable logistic regression analyses were performed. Sixteen respondents (1.2%) stated “non-binary” for gender and, because of the low number, were not included in comparative analyses. Due to limited diversity in the study population, the race/ethnicity variable was dichotomized into "non-Hispanic White" and "other," introducing significant heterogeneity within the latter group. This approach, however, facilitated the inclusion of the variable in the data analysis. Regarding helmet importance, comparison of the median was performed instead of the mean given the asymmetry of the data, and non-parametric tests, the Wilcoxon rank sum test or Kruskal–Wallis test depending on number of groups tested, was utilized. All *p*-values were two-tailed, and a value < 0.05 was considered statistically significant. Missing data were not included in analyses.

## Results

1331 adolescent FFA members (12–18 years) participated in the study. See Table 1. Just over two-fifths were male and more than two-thirds were 15–17 years. One half lived on a farm, about one-fifth lived in the country but not on a farm and more than a quarter were from towns. The vast majority (96%) were non-Hispanic White.

**Table 1:** Demographic variables of survey respondents at the 2022 Iowa FFA Leadership Conference

	*n* (col %)^a^
Group *N*	1331
*Sex*	
Male	543 (41)
Female	770 (58)
Nonbinary	16 (1.2)
Other	0 (0)
*Age*	
13 years	66 (5)
14 years	171 (13)
15 years	337 (26)
16 years	300 (23)
17 years	272 (21)
18 years	166 (13)
*Residence*	
Farm	670 (50)
Country/not farm	285 (21)
Town	376 (28)
*Race*	
Non-Hispanic White	1320 (96)
Black/African American	17 (1)
Asian	8 (0.5)
Latino/Latinx	35 (3)
Other	13 (1)

^a^The sum of* n* may not equal the total group *N* due to missing values

### Ownership of ATVs, dirt bikes and motorcycles

ATV ownership by respondent families was significantly higher than that for dirt bikes or motorcycles. See Table 2. For all three vehicles, male respondents had higher proportions that reported owning them than females. Those that lived on a farm or in the country/not a farm as compared to those from towns and non-Hispanic Whites as compared to those of other races/ethnicities had higher percentages owning ATVs and dirt bikes. Farm residents had the highest ownership of ATVs. Logistic regression analysis controlling for the other variables in Table 2 (data not shown) indicated the odds of non-Hispanic White families owning ATVs was 4.0 times (95% CI 1.3–11.7) greater than that of other races/ethnicities. In addition, respondents from farms and the country/not a farm had 5.0 (95% CI 2.3–10.6) and 2.5 (95% CI 1.2–5.7) times higher odds, respectively, of owning ATVs versus town residents.

**Table 2:** Contingency table analyses regarding whether the families of 2022 Iowa FFA Leadership Conference survey respondents owned a motorcycle, ATV or dirt bike

Contingency table analyses									
	Motorcycle			ATV			Dirt bike		
	Owned	Did not own	*p* value	Owned	Did not own	*p* value	Owned	Did not own	*p* value
	*n* (row %)^a^	*n* (row %)^a^		*n* (row %)^a^	*n* (row %)^a^		*n* (row %)^a^	*n* (row %)^a^	
*All*	291 (22)	1029 (78)		833 (65)	445 (35)		381 (29)	930 (71)	
*Sex*									
Male	136 (25)	403 (75)	0.02	368 (71)	152 (29)	< 0.001	183 (34)	352 (66)	< 0.001
Female	150 (20)	613 (80)		456 (61)	286 (39)		192 (25)	566 (75)	
*Age*									
16–18 yrs	155 (21)	575 (79)	0.656	472 (66)	239 (34)	0.361	215 (30)	512 (70)	0.863
13–15 yrs	128 (22)	443 (78)		350 (64)	199 (36)		164 (29)	402 (81)	
*Residence*									
Farm	137 (21)	526 (79)	0.42	500 (78)	140 (22)	< 0.001	210 (32)	448 (68)	< 0.001
Country^b^	69 (24)	214 (76)		182 (67)	91 (33)		99 (35)	181 (65)	
Town	85 (23)	289 (77)		151 (41)	214 (59)		72 (19)	301 (81)	
*Race*									
NH White	275 (22)	971 (78)	1.0	813 (67)	397 (33)	< 0.001	369 (30)	869 (70)	0.024
Other^c^	16 (22)	57 (78)		20 (30)	47 (70)		12 (17)	60 (83)	

*ATV* all-terrain vehicle, *yrs* years, *NH White* non-Hispanic White

^a^The sum of* n* for a variable may not equal the total group *N* due to missing values

^b^Respondents who live in the country, but not on a farm

^c^Respondents who were races and ethnicities other than non-Hispanic White

### Ridden an ATV, dirt bike and/or motorcycle in the past year

Around twice the proportion of participants reported riding an ATV in the past year as compared to dirt bikes and motorcycles. See Table 3. Similar to ownership, higher percentages of males had ridden each of the three vehicles as compared to females. Still, 70% of females had ridden an ATV in the past year. Greater percentages of participants from farms and from the country/not a farm had ridden an ATV and a dirt bike in the past year as compared to those that lived in towns. Non-Hispanic Whites also had higher proportions that had ridden an ATV in past year versus other races/ethnicities. Those whose families owned each of the vehicles had much higher proportions having ridden them in the past year as compared to non-owners. Logistic regression analysis demonstrated males had odds 2.3 (95% CI 1.1–4.9) times greater to have ridden a dirt bike as females (data not shown).

**Table 3:** Contingency table analyses regarding whether 2022 Iowa FFA Leadership Conference survey respondents had ridden a motorcycle, ATV or dirt bike in the past year

Contingency table analyses									
	Motorcycle			ATV			Dirt bike		
	Ridden	Never ridden	*p* value	Ridden	Never ridden	*p* value	Ridden	Never ridden	*p* value
	*n* (row %)^a^	*n* (row %)^a^		*n* (row %)^a^	*n* (row %)^a^		*n* (row %)^a^	*n* (row %)^a^	
*All*	406 (31)	896 (69)		979 (77)	293 (23)		516 (40)	778 (60)	
*Sex*									
Male	208 (38%)	335 (62)	< 0.001	427 (79)	116 (21)	< 0.001	265 (49%)	278 (51)	< 0.001
Female	187 (24%)	583 (76)		539 (70)	231 (30)		242 (31%)	528 (69)	
*Age*									
16–18 years	220 (30)	518 (70)	0.541	551 (75)	187 (25)	0.448	271 (37)	467 (63)	0.091
13–15 years	181 (32)	393 (68)		417 (73)	157 (27)		238 (41)	336 (59)	
*Residence*									
Farm	183 (27)	487 (73)	0.037	533 (80)	137 (20)	< 0.001	266 (40)	404 (60)	< 0.001
Country^b^	98 (34%)	187 (66)		218 (76)	67 (24)		132 (46)	153 (54)	
Town	125 (33%)	251 (67)		228 (61)	148 (39)		118 (31)	258 (69)	
*Race*									
NH White	384 (31)	873 (69)	1.0	951 (76)	306 (24)	< 0.001	495 (39)	762 (61)	0.092
Other^c^	22 (30)	51 (70)		28 (38)	45 (62)		21 (40)	52 (60)	
*Ownership*									
Yes	203 (70)	88 (30)	< 0.001	800 (96)	33 (4)	< 0.001	330 (87)	51 (13)	< 0.001
No	202 (20)	827 (80)		179 (40)	266 (60)		186 (20)	744 (80)	

*ATV* all-terrain vehicle, *yrs* years, *NH White* non-Hispanic White

^a^The sum of* n* for a variable may not equal the total group *N* due to missing values

^b^Respondents who live in the country, but not on a farm

^c^Respondents who were races and ethnicities other than non-Hispanic White

### Frequency of riding ATVs, dirt bikes and motorcycles in the past year

Of participants that reported riding these vehicles in the past year, higher proportions of ATV riders rode at least weekly as compared to riders of dirt bikes and motorcycles. See Table 4. Males had higher proportions who rode dirt bikes and motorcycles at least weekly as compared to females, but similar percentages of male and female ATV riders rode at least weekly. Older teens as compared to younger teens and participants from farms and the country/not farms as compared to those from towns had higher proportions of riding ATVs at least weekly. Respondents whose families owned the vehicle had much higher percentages riding them at least weekly versus non-owners. Logistic regression analysis showed males had 2.9 (95% CI 1.3–6,1), 3.3 (95% CI 1.3–8.5) and 5.9 (95% CI 1.8–19.5) times higher odds of riding ATVs, dirt bikes and motorcycles, respectively, at least weekly as compared with females (data not shown). Participants from farms had 2.5 (95% CI 1.1–5.8) times greater odds of riding ATVs at least weekly versus those from towns.

**Table 4:** Contingency table analyses regarding the frequency 2022 Iowa FFA Leadership Conference survey respondents had ridden a motorcycle, ATV or dirt bike in the past year

Contingency table analyses									
	Motorcycle			ATV			Dirt bike		
	Few times a year/monthly	Weekly/daily	*p* value	Few times a year/monthly	Weekly/daily	*p* value	Few times a year/monthly	Weekly/daily	*p* value
	*n* (row %)^a^	*n* (row %)^a^		*n* (row %)^a^	*n* (row %)^a^		*n* (row %)^a^	*n* (row %)^a^	
*All*	343 (84)	63 (16)		517 (51)	500 (49)		364 (71)	152 (29)	
*Sex*									
Male	160 (77)	48 (23)	< 0.001	159 (37)	268 (63)	0.525	164 (62%)	101 (38)	0.005
Female	147 (93)	11 (7)		148 (40)	225 (60)		138 (75%)	46 (25)	
*Age*									
16–18 yrs	186 (85)	34 (15)	1.0	116 (30)	276 (70)	< 0.001	133 (63%)	78 (37)	0.225
13–15 yrs	153 (85)	28 (15)		201 (48)	216 (52)		164 (69%)	74 (31)	
*Residence*									
Farm	154 (84)	29 (16)	0.956	197 (37)	336 (63)	< 0.001	183 (69%)	83 (31)	0.595
Country^b^	67 (83)	14 (17)		64 (42)	88 (58)		69 (66%)	36 (34)	
Town	105 (84)	20 (16)		152 (67)	76 (33)		85 (72%)	33 (28)	
*Race*									
NH White	328 (85)	56 (15)	0.061	461 (48)	490 (52)	1.0	352 (71%)	143 (29)	0.054
Other^c^	15 (68)	7 (32)		9 (47)	10 (53)		8 (47%)	9 (53)	
*Ownership*									
Yes	149 (73)	54 (27)	< 0.001	323 (40)	477 (60)	< 0.001	190 (58%)	140 (42)	< 0.001
No	193 (96)	9 (4)		156 (85)	23 (15)		174 (94%)	12 (6)	

*ATV* all-terrain vehicle, *yrs* years, *NH White* non-Hispanic White

^a^The sum of* n* for a variable may not equal the total group *N* due to missing values

^b^Respondents who live in the country, but not on a farm

^c^Respondents who were races and ethnicities other than non-Hispanic White

### Helmet use on ATVs, dirt bikes and motorcycles

Higher proportions of participants stated they always/most of the time wore a helmet when riding motorcycles and dirt bikes as compared to ATVs. See Table 5. Moreover, higher proportions of participants stated they never wore helmets riding ATVs (49%) as compared to motorcycles (21%) and dirt bikes (21%), *p* < 0.001 for both (data not shown in a table). Females had higher proportions stating they always/mostly wore helmets on motorcycles but were not different from males for ATVs and dirt bikes. Overall, those who lived on farms had lower helmet use for all three vehicles versus those who lived elsewhere, *p* < 0.04 for all three comparisons (data not in table), with only 15% reporting always/mostly wearing helmets when on ATVs.

**Table 5:** Contingency table analyses regarding the frequency 2022 Iowa FFA leadership conference survey respondents wore a helmet while riding a motorcycle, ATV or dirt bike

Contingency table analyses									
	Motorcycle			ATV			Dirt bike		
	Always/mostly	Never/rarely/sometimes	*p* value	Always/mostly	Never/rarely/sometimes	*p* value	Always/mostly	Never/rarely/sometimes	*p* value
	*n* (row %)^a^	*n* (row %)^a^		*n* (row %)^a^	*n* (row %)^a^		*n* (row %)^a^	*n* (row %)^a^	
*All*	232 (58)	170 (42)		206 (21)	766 (79)		261 (51)	248 (49)	
*Sex*									
Male	103 (50)	101 (50)	0.008	98 (23)	327 (77)	0.23	131 (50)	133 (50)	0.452
Female	119 (64)	66 (36)		105 (20)	429 (80)		126 (53)	110 (47)	
*Age*									
16–18 years	120 (55)	97 (45)	0.257	100 (25)	308 (75)	0.008	136 (51)	133 (49)	0.789
13–15 years	110 (61)	69 (39)		106 (19)	447 (81)		121 (52)	111 (48)	
*Residence*									
Farm	88 (49)	92 (51)	0.003	80 (15)	446 (85)	< 0.001	123 (46)	142 (54)	0.073
Country^b^	66 (69)	29 (31)		66 (30)	152 (70)		71 (55)	58 (45)	
Town	77 (62)	48 (38)		60 (36)	168 (63)		66 (58)	48 (42)	
*Race*									
NH White	217 (57)	162 (43)	0.533	200 (21)	744 (79)	1.0	252 (52)	236 (48)	0.428
Other	14 (67)	7 (33)		6 (21)	22 (79)		8 (40)	12 (60)	
*Ownership*									
Yes	124 (63)	77 (37)	0.122	164 (21)	630 (79)	0.444	183 (56)	143 (44)	0.004
No	106 (54)	92 (46)		42 (24)	136 (76)		77 (42)	105 (58)	
*Riding frequency*									
Monthly/few	195 (58)	143 (42)	1.0	115 (24)	361 (76)	0.033	174 (49)	184 (51)	0.089
Daily/weekly	36 (58)	26 (42)		91 (18)	405 (82)		86 (57)	64 (43)	
*Strict helmet rule*^d^									
Yes	42 (75)	14 (25)	0.003	111 (62)	67 (38)	< 0.001	67 (74)	24 (26)	< 0.001
No	139 (52)	127 (48)		93 (12)	695 (88)		155 (45)	193 (55)	

*ATV* all-terrain vehicle, *NH White* non-Hispanic White

^a^The sum of* n* for a variable may not equal the total group *N* due to missing values

^b^Respondents who live in the country, but not on a farm

^c^Respondents who were races and ethnicities other than non-Hispanic White

^d^Respondent’s family had a strict ATV “no helmet, no riding” rule. For motorcycles and dirt bikes, this only includes those that were also ATV riders

Owners of dirt bikes had higher proportions using helmets always/mostly versus non-owners which was not true related to motorcycles and ATVs. More frequent ATV riders (at least weekly) had lower percentages always/mostly using helmets as compared to less frequent riders. For ATV riders whose parents had a strict “No Helmet, No Riding” rule, 62% (111/178) stated they always/mostly wore a helmet which was far higher than any other demographic group. In fact, those with strict helmet rules had odds 12.1 (95% CI 5.1–28.8) times greater of using helmets always/mostly as compared to those without such a rule (data not shown). In addition, those with a strict ATV helmet rule also had greater helmet use when riding dirt bikes (74%) and motorcycles (75%) than those that did not have that rule.

### Helmet laws for ATVs, dirt bikes and motorcycles

A greater proportion of respondents supported laws requiring helmets for riding motorcycles as compared to dirt bikes, and they supported helmet laws for motorcycles and for dirt bikes by greater percentages than ATVs. See Table 6. Females had higher percentages supporting helmet laws then males for all three vehicles. Those living on farms had less support for helmet laws than those living elsewhere. Owners and riders of all three vehicles had lower proportions supporting helmet requirement laws than non-owners and non-riders, respectively. Respondents whose families had a strict “No Helmet, No Riding” rule had the highest proportion supporting helmet laws for ATVs (nearly two-thirds), greater than any other demographic group. Logistic regression analysis showed that those with strict helmet rules had 10.0 (95% CI 4.2–23.8) times higher odds of supporting an ATV helmet law versus those without a rule (data not shown).

**Table 6:** Contingency table analyses regarding whether 2022 Iowa FFA Leadership Conference survey respondents believed there should be a law requiring helmet use while riding a motorcycle, ATV or dirt bike

Contingency table analyses									
	Motorcycle			ATV			Dirt bike		
	Law	No law	*p* value	Law	No law	*p* value	Law	No law	*p* value
	*n* (row %)^a^	*n* (row %)^a^		*n* (row %)^a^	*n* (row %)^a^		*n* (row %)^a^	*n* (row %)^a^	
*All*	942 (72)	360 (28)		382 (31)	862 (69)		724 (56)	562 (44)	
*Sex*									
Male	304 (57)	227 (43)	< 0.001	126 (25)	379 (75)	< 0.001	222 (42)	303 (58)	< 0.001
Female	625 (83)	128 (17)		247 (34)	476 (66)		492 (66)	252 (34)	
*Age*									
16–18 years	489 (68)	229 (32)	< 0.001	191 (28)	501 (72)	0.012	390 (55)	322 (45)	0.188
13–15 years	441 (78)	125 (22)		184 (34)	350 (66)		326 (59)	230 (41)	
*Residence*									
Farm	452 (69)	204 (31)	0.018	155 (25)	470 (75)	< 0.001	345 (53)	304 (47)	0.037
Country^b^	206 (75)	69 (25)		88 (33)	182 (67)		154 (57)	117 (43)	
Town	284 (77)	87 (23)		139 (40)	210 (60)		225 (61)	141 (39)	
*Race*									
NH White	891 (72)	338 (28)	0.669	358 (30)	824 (70)	0.279	685 (56)	532 (44)	1.0
Other^c^	50 (69%)	22 (31)		23 (38)	38 (62)		38 (56)	30 (44)	
*Ownership*									
Yes	180 (63)	108 (37)	< 0.001	199 (24)	615 (76)	< 0.001	163 (44)	211 (56)	< 0.001
No	761 (75)	252 (25)		183 (43)	247 (57)		561 (62)	351 (38)	
*Ridden in past year*									
Yes	242 (61)	157 (39)	< 0.001	235 (25)	720 (75)	< 0.001	212 (42)	293 (58)	< 0.001
No	691 (78)	199 (22)		145 (51)	141 (49)		506 (66)	266 (34)	
*Riding frequency*									
Monthly/few	216 (64)	121 (36)	0.002	126 (27)	339 (73)	0.842	156 (44)	201 (56)	0.265
Daily/weekly	26 (42)	36 (58)		109 (28)	281 (72)		56 (38)	92 (62)	
*Strict helmet rule*^d^									
Yes	–	–	–	112 (64)	63 (36)	< 0.001	–	–	
No	–	–	–	124 (16)	653 (84)		–	–	

*ATV* all-terrain vehicle, *NH White* non-Hispanic White

^a^The sum of* n* for a variable may not equal the total group *N* due to missing values

^b^Respondents who live in the country, but not on a farm

^c^Respondents who were races and ethnicities other than non-Hispanic White

^d^Respondent’s family had a strict ATV “no helmet, no riding” rule

### Helmet use importance

Helmet use importance (rated from 1 to 10, 10 high) were not different between motorcycles (mean 8.6, median 10) and dirt bikes (mean 8.3, median 10), but much lower for ATVs (mean 6.1, median 6). See Table 7. Females, non-owners, and those supporting helmet laws all had higher helmet use importance ratings as compared to their peers. There was a difference in the level of importance ascribed to helmet use for all three vehicles based on helmet use frequency. Those whose families had a strict ATV “No Helmet, No Riding” rule had higher helmet importance (median 9) than those with no such rule (median 5).

**Table 7:** Comparisons of the median importance ascribed by 2022 Iowa FFA Leadership Conference survey respondents to helmet use while riding a motorcycle, ATV or dirt bike

	Median comparisons of helmet use importance					
	Motorcycle		ATV		Dirt Bike	
	Median importance	*p* value	Median importance	*p* value	Median importance	*p* value
	(1–10)^a^		(1–10)^a^		(1–10)^a^	
*All*	10		6		10	
*Sex*						
Male	9	< 0.001	6	< 0.001	9	< 0.001
Female	10		7		10	
*Age*						
16–18 years	10	0.211	6	0.001	10	0.574
13–15 years	10		7		9	
*Residence*						
Farm	10	0.326	5	0.065	9	0.561
Country but not on a farm	10		7		10	
Town	10		7		10	
*Race*						
NH White	10	0.101	6	0.033	9	0.018
Other races^b^	10		7		10	
*Ownership*						
Yes	9	< 0.001	5	< 0.001	9	< 0.001
No	10		8		10	
*Ridden in past year*						
Yes	10	0.069	6	0.067	9	0.111
No	10		10		10	
*Riding frequency*						
Monthly/few times a year	9	0.003	6	< 0.001	9	0.099
Daily/weekly	8		5		8	
*Helmet use*						
Always	10	< 0.001	10	< 0.001	10	< 0.001
Mostly	9		8		8	
Sometimes/rare	7		6		7	
Never	5		4		5	
*Helmet law*						
Support	10	< 0.001	10	< 0.001	10	< 0.001
Against	8		5		7	
*Strict “no helmet, no ride” rule*^c^						
Yes	–		9	< 0.001	–	
No	–		5		–	

*ATV* all-terrain vehicle, *NH White* non-Hispanic White

^a^Median importance was 1–10, with 1 being “not at all important” and 10 being “extremely important”

^b^Respondents who were races and ethnicities other than non-Hispanic White

^c^Respondent’s family had a strict ATV “no helmet, no riding” rule

## Discussion

A significant proportion of adolescents in the study were exposed to ATVs, motorcycles and dirt bikes, and helmet use (always/most of the time) was not greater than 58% for any of the vehicles. Ownership was highest for ATVs (65%) and more than three-quarters of all had ridden an ATV in the past year. While over half of riders reported using helmets always or most of the time on motorcycles and dirt bikes, this was only 21% for ATVs. The mean importance of helmet use and their level of support for helmet laws mirrored helmet use for each vehicle. In addition, the greater the importance participants ascribed to helmet use for a vehicle, the more frequently they wore a helmet when riding.

Respondents from farms as compared to those living elsewhere had the lowest helmet use and the least support of helmet laws for all three vehicles, even though their median rating of the importance of helmet use was not significantly different from their peers. Similar to adolescents from farms in our study, farmers have reported low ATV helmet use (Irwin et al. 2022; Mcintosh et al. 2016; Jennissen et al. 2017). Specifically, a study of attendees of the 2012 and 2013 Farm Progress Show, the largest U.S. outdoor farm show, found that among respondents, farmers had the lowest ATV helmet use with nearly three-fifths (58%) stating they never/almost never wore a helmet (Jennissen et al. 2017). Other studies have demonstrated low helmet use on ATVs among adolescents from farms (Hafner et al. 2010; Burgus et al. 2009; Goldcamp et al. 2006; Jinnah and Stoneman 2016).

Helmet use and the importance ascribed to wearing a helmet was much lower for ATVs than dirt bikes and motorcycles in the study. However, research has shown that pediatric ATV crash victims have a relatively high morbidity and mortality (Collins et al. 2007; Nabaweesi et al. 2018; Linnaus et al. 2017; Elzaim et al. 2022; Shults et al. 2005; Acosta and Rodriguez 2003). In fact, the severity of injuries on ATVs is more comparable to motor vehicle collisions than that of other sports and recreational activities (Nabaweesi et al. 2018). One study found that youth in ATV crashes were 7 times more likely to be hospitalized than other trauma causes and twice as likely as patients in motor vehicle crashes (Shults et al. 2005). In addition, the proportion of head injuries is higher in ATV-related crashes than with motorcycles (Collins et al. 2007; Linnaus et al. 2017; Acosta and Rodriguez 2003). Studies have shown many parents and adolescents do not consider driving ATVs to be dangerous and perceive the risk of serious injury to be low (Adams et al. 2013; Wymore et al. 2020). Undoubtedly, these distorted beliefs are likely factors in the low use of helmets on ATVs. Although few children and adolescents receive formal ATV training, this training has been positively associated with increased helmet use (Burgus et al. 2009; Jennissen et al. 2022).

There are no helmet laws in Iowa except the requirement to use them in public off-highway vehicle parks (Institute and for Highway Safety ((IIHS), Highway Loss Data Institute (HLDI) 2023; Iowa Department of Natural Resources. Off-highway vehicle reference guide 2024). Over half of the study FFA members supported laws mandating helmet use for dirt bikes and motorcycles, whereas ATV helmet law support was less than one-third, even though the risk for head injury is similar. Universal helmet laws covering all riders have been shown to be the most effective way to increase helmet use (NHTSA 2009; Houston and Richardson 2008) and are associated with a 36–45% decline in motorcycle crash mortality (Nortica et al. 2020 Nov). Nineteen states and the District of Columbia have universal laws (Institute and for Highway Safety ((IIHS), Highway Loss Data Institute (HLDI). 2023). Partial laws covering certain ages (usually those that are younger) have not been effective (NHTSA 2009; Houston and Richardson 2008; Nortica et al. 2020).

Nearly all states had universal laws by the early 1970’s after they were required to enact helmet legislation in order to qualify for Federal funding related to highway construction and some safety programs (NHTSA 2019). However, this requirement was removed in 1976 and in the subsequent four years almost half the states had repealed their universal laws. An immediate increase in motorcycle fatalities was seen as exemplified by Michigan, one of the most recent to change its law in 2013, which experienced an 18% increase in motorcycle fatalities (NHTSA 2019). Similarly, states with more ATV safety laws have lower fatality rates, including for children (Helmkamp 2001; Helmkamp et al. 2012). One of the most effective way for states to save motorcycle, dirt bike and ATV rider lives is the passage and enforcement of universal helmet laws.

Over 60% of study participants who reported a strict ATV “No Helmet, No Riding” rule in their family wore a helmet always or most of the time when riding ATVs. Although there is still room for improvement, this proportion was by far higher than that of any other demographic group. We did not ask participants whether they had “No Helmet, No Riding” rules for other vehicles, but those who had strict ATV rules were also found to have higher helmet use on motorcycles and dirt bikes. In addition, the rating of helmet importance was much higher for those with a strict ATV helmet rule (median 9) as compared to those without (median 5).

Previous studies have shown that firm rules requiring helmet use are critical to helmet wearing by children (Miller et al. 1996; Berg and Westerling 2001; Khambalia et al. 2005; Keezer et al. 2007). One study found that a strict rule increased the likelihood of bicycle helmet use 46-fold (Miller et al. 1996). Parents stated in another study that the most effective method for getting their children to wear helmets on bicycles and ATVs was a non-negotiable “No Helmet, No Riding” rule (Wymore et al. 2020). Adolescents shared in a focus group that the main reason they wore ATV helmets was that their riding club or parents mandated it (Adams et al. 2013). Even adolescents, whose adherence to rules at times may be lacking, have higher proportions that use helmets when parents have strict requirements than when none exist (Berg and Westerling 2001). Parents should be encouraged to start helmet use early, be good helmet-wearing role models, and to implement a non-negotiable “No Helmet, No Riding” rule that is strictly enforced with negative consequences (e.g., riding privileges revoked for a week) for non-compliance.

### Limitations

Our study was performed in a single Midwestern state with a population primarily rural and non-Hispanic White. Thus, our findings may not be generalizable to other states, urban settings or areas of greater racial/ethnic diversity. Moreover, comparisons by race/ethnicity should be interpreted with caution given the category “Other” was quite small and diverse. We also used a convenience sample of FFA members that were mostly from rural areas, so our results may not be representative of adolescents across the entire state. However, the vast majority of Iowa counties were represented in the sample. Data collected was self-reported so is likely subject to recall bias and social desirability. Surveys were anonymous and completed independently which should have decreased the social desirability effect.

## Conclusions

ATV ownership and use by adolescents in the study was extremely common, especially those from farms. The importance of wearing a helmet while riding motorcycles and dirt bikes was much higher than for ATVs, and helmet use mirrored their importance rating. Farm youth had lower proportions wearing helmets for all vehicles and less support for laws mandating helmet use. Whereas, over one-half of study participants supported a helmet law for motorcycles and dirt bikes. Respondents whose families had an ATV “No Helmet, No Riding” rule had higher ratings of helmet importance and more frequent helmet use than those without a strict rule. Our study indicates that the safety culture surrounding helmet use is relatively poor among rural adolescents, especially on farms, and deserves targeted interventions. The passage and enforcement of universal helmet laws is one of the many essential ways of improving the safety culture surrounding these vehicles and thereby preventing deaths and injuries among rural adolescents who ride them.

## Data Availability

Data and materials are available to other parties for research purposes after a data sharing agreement plan is agreed to and signed. Those interested should contact the corresponding author.

## Abbreviations

ATV: All-terrain vehicle
DOT: Department of Transportation
ED: Emergency department
e.g.: Exempli gratia (for example)
FFA: Formerly Future Farmers of America
ICU: Intensive care unit
NHTSA: National Highway Traffic Safety Administration
NOPUS: National Occupant Protection Use Survey
SFCH: Stead Family Children’s Hospital
U.S.: United States

## References

[CR1] Abdelgawad AA, Maxfield D, Tran S, Mclean S, Kanlic EM. Dirt bikes injuries in children. Musculoskelet Surg. 2013;97(3):211–5. 23918697 10.1007/s12306-013-0298-4

[CR2] Acosta JA, Rodriguez P. Morbidity associated with four-wheel all-terrain vehicles and comparison with that of motorcycles. J Trauma. 2003;55:282–4. 12913638 10.1097/01.TA.0000080525.77566.ED

[CR3] Adams LE, Aitken ME, Mullins SH, Miller BK, Graham J. Barriers and facilitators to all-terrain vehicle helmet use. J Trauma Acute Care Surg. 2013;75(4 Suppl 3):S296–300. 23702632 10.1097/TA.0b013e318292421f

[CR5] Barron S, Falank C, Ontengco J, Chung B, Carter DW. Severity and patterns of injury in helmeted vs. non-helmeted motorcyclists in a rural state. J Safety Res. 2021;77:212–6. 34092311 10.1016/j.jsr.2021.03.004

[CR6] Berg P, Westerling R. Bicycle helmet use among school children–the influence of parental involvement and children’s attitudes. Inj Prev. 2001;7(3):218–22. 11565988 10.1136/ip.7.3.218 PMC1730748

[CR7] Bhutta ST, Greenberg SB, Fitch SJ, Parnell D. All-terrain vehicle injuries in children: injury patterns and prognostic implications. Pediatr Radiol. 2004;34(2):130–3. 14605783 10.1007/s00247-003-1085-4

[CR8] Blincoe, LJ, Miller TR, Zaloshnja E, Lawrence BA. The economic impact of motor vehicle crashes. 2010. (revised). May 2015 Revised. National Highway Traffic Safety Administration, Washington, DC. (Publication No. DOT HS 812 013). Available at: https://crashstats.nhtsa.dot.gov/Api/Public/ViewPublication/812013 Accessed 2 Feb 2024.

[CR9] Bowman SM, Aitken ME. Still unsafe, still in use: ongoing epidemic of allterrain vehicle injury hospitalizations among children. J Trauma. 2010;69(6):1344–2134. 20962681 10.1097/TA.0b013e3181ea283d

[CR10] Bowman SM, Aitken ME, Helmkamp JC, Maham SA, Graham CJ. Impact of helmets on injuries to riders of all-terrain vehicles. Inj Prev. 2009;15(1):3–7. 19190268 10.1136/ip.2008.019372

[CR11] Boyle L. Motorcycle helmet use in 2022-overall results. Aug 2023. Traffic Safety Facts Research Note. (Report No. DOT HS 813 505). National Highway Traffic Safety Administration. Available at: https://crashstats.nhtsa.dot.gov/Api/Public/ViewPublication/813505 Accessed 2 Feb 2024.

[CR12] Brown RL, Koepplinger ME, Mehlman CT, Gittelman M, Garcia VF. All-terrain vehicle and bicycle crashes in children: epidemiology and comparison of injury severity. J Pediatr Surg. 2002;37(3):375–80. 11877651 10.1053/jpsu.2002.30826

[CR13] Burgus SK, Madsen MD, Sanderson WT, Rautiainen RH. Youths operating all-terrain vehicles–implications for safety education. J Agromed. 2009;14(2):97–104. 10.1080/10599240902751047 19437264

[CR14] Carr AM, Bailes JE, Helmkamp JC, Rosen CL, Miele VJ. Neurological injury and death in all-terrain vehicle crashes in West Virginia: a 10-year retrospective review. Neurosurgery. 2004;54(4):861–6. 15046651 10.1227/01.NEU.0000114922.46342.38

[CR15] Centers for Disease Control and Prevention (CDC). Nonfatal injuries from off-road motorcycle riding among children and teens—United States, 2001–2004. MMWR Morb Mortal Wkly Rep. 2006 9;55(22):621–4. 16760889

[CR16] Coben JH, Steiner CA, Miller TR. Characteristics of motorcycle-related hospitalizations: comparing states with different helmet laws. Accid Anal Prev. 2007;39(1):190–6. 16920053 10.1016/j.aap.2006.06.018

[CR17] Collins CL, Smith GA, Comstock RD. Children plus all nonautomobile motorized vehicles (not just all-terrain vehicles) equals injuries. Pediatrics. 2007;120(1):134–41. 17606570 10.1542/peds.2006-3612

[CR18] Denning GM, Jennissen CA. All-terrain vehicle fatalities on paved roads, unpaved roads, and off-road: evidence for informed roadway safety warnings and legislation. Traffic Inj Prev. 2016;17(4):406–12. 26065484 10.1080/15389588.2015.1057280

[CR19] Denning G, Jennissen C. Pediatric and adolescent injury in all-terrain vehicles. Res Sports Med. 2018;26(S1):38–56. 30431365 10.1080/15438627.2018.1438279

[CR20] Denning G, Harland K, Ellis D, Jennissen C. More fatal all-terrain vehicle crashes occur on the roadway than off: Increased risk-taking characterises roadway fatalities. Inj Prev. 2013a;19:250–6. 23257569 10.1136/injuryprev-2012-040548 PMC3717765

[CR21] Denning G, Jennissen C, Harland K, Ellis D, Buresh C. All-terrain vehicles (ATVs) on the road: a serious traffic safety and public health concern. Traffic Inj Prev. 2013b;14(1):78–85. 23259522 10.1080/15389588.2012.675110

[CR22] Denning G, Harland K, Jennissen C. Age-based risk factors for pediatric ATV-related fatalities. Pediatrics. 2014;134(6):1094–102. 25422012 10.1542/peds.2014-1993

[CR23] Elzaim JS, Vatcheva K, Torres-Reveron A, Pequeno G, Betancourt-Garcia MM. Comparative analysis of all-terrain vehicles, motorcycle and automobile-related trauma in a rural border community of the USA. BMJ Open. 2022;12(9):e054289. 36302559 10.1136/bmjopen-2021-054289 PMC9558800

[CR24] Ganga A, Kim EJ, Araia ES, Hagan M, Shao B, Svokos K, Klinge PM, Cielo DJ, Fridley JS, Gokaslan ZL, Toms SA, Sullivan PZ. Pediatric all-terrain vehicle (ATV) related head injury rates and patterns: a 10-year nationwide analysis. Am J Emerg Med. 2023;67:56–62. 36804750 10.1016/j.ajem.2023.02.007

[CR25] GAO. All-terrain vehicles: how they are used, crashes, and sales of adult-sized vehicles for children’s use. 2010 report to congressional committees (GAO-10–418). Available at: www.gao.gov/new.items/d10418.pdf. Accessed 3 Feb 2024.

[CR26] Gerberich SG, Gibson RW, French LR, et al. Injuries among children and youth in farm households: regional Rural injury study-I. Inj Prev. 2001;7(2):117–22. 11428558 10.1136/ip.7.2.117 PMC1730710

[CR27] Gittelman MA, Pomerantz WJ, Groner JI, Smith GA. Pediatric all-terrain vehicle-related injuries in Ohio from 1995 to 2001: using the injury severity score to determine whether helmets are a solution. Pediatrics. 2006;117(6):2190–5. 16740864 10.1542/peds.2005-2603

[CR28] Goldcamp EM, Myers J, Hendricks K, Layne L, Helmkamp J. Nonfatal all-terrain vehicle-related injuries to youths living on farms in the United States, 2001. J Rural Health. 2006;22(4):308–13. 17010027 10.1111/j.1748-0361.2006.00051.x

[CR29] Hafner JW, Hough SM, Getz MA, Whitehurst Y, Pearl RH. All-terrain vehicle safety and use patterns in central Illinois youth. J Rural Health. 2010;26(1):67–72. 20105270 10.1111/j.1748-0361.2009.00267.x

[CR30] Helmkamp JC. A comparison of state-specific all-terrain vehicle-related death rates, 1990–1999. Am J Public Health. 2001;91(11):1792–5. 11684604 10.2105/AJPH.91.11.1792 PMC1446879

[CR31] Helmkamp JC, Furbee PM, Coben JH, Tadros A. All-terrain vehicle-related hospitalizations in the United States, 2000–2004. Am J Prev Med. 2008;34(1):39–45. 18083449 10.1016/j.amepre.2007.09.016

[CR32] Helmkamp JC, Aitken ME, Lawrence BA. ATV and bicycle deaths and associated costs in the United States, 2000–2005. Public Health Rep. 2009;124(3):409–18. 19445417 10.1177/003335490912400310 PMC2663877

[CR33] Helmkamp JC, Aitken ME, Graham J, Campbell CR. State-specific ATV-related fatality rates: an update in the new millennium. Public Health Rep. 2012;127(4):364–74. 22753979 10.1177/003335491212700404 PMC3366374

[CR34] Hendricks KJ, Layne LA, Goldcamp EM, Myers JR. Injuries to youth living on U.S. farms in 2001 with comparison to 1998. J Agromed. 2005;10(4):19–26. 10.1300/J096v10n04_05 16702120

[CR35] Ho CV, Dunne JR, Stroud WR, Fonseca AH, Davis FE, Bromberg WJ. Analysis of all-terrain vehicle trauma data: implications for increased regulation and injury prevention. Am Surg. 2017;83(4):348–53. 28424128 10.1177/000313481708300420

[CR36] Holt MF, Fortmann J, Testerman GM. Trauma surgeon-led and funded injury prevention program decreases number of all-terrain vehicle-related admissions. Am Surg. 2022;88(4):638–42. 34978213 10.1177/00031348211050815

[CR37] Houston DJ, Richardson LE. Motorcyclist fatality rates and mandatory helmet-use laws. Accid Anal Prev. 2008;40:200–8. 18215549 10.1016/j.aap.2007.05.005

[CR38] Humphries RL, Stone CK, Stapczynski JS, Florea S. An assessment of pediatric all-terrain vehicle injuries. Pediatr Emerg Care. 2006;22(7):491–4. 16871109 10.1097/01.pec.0000227383.69014.36

[CR39] Insurance Institute for Highway Safety ((IIHS)/Highway Loss Data Institute (HLDI). Fatality facts 2021: motorcycles and ATVs. May 2023. Available at: https://www.iihs.org/topics/fatality-statistics/detail/motorcycles-and-atvs Accessed 17 Jan 2024.

[CR40] Insurance Institute for Highway Safety ((IIHS)/Highway Loss Data Institute (HLDI). Motorcycle helmet use laws. February 2024. Available at: https://www.iihs.org/topics/motorcycles/motorcycle-helmet-laws-tables.org) Accessed 2 Feb 2024.

[CR41] Iowa Department of Natural Resources. Off-highway vehicle reference guide. Available at: https://www.iowadnr.gov/portals/idnr/uploads/atv/ohvreferenceguide.pdf Accessed 2 Feb 2024.

[CR42] Iowa FFA Association. About Iowa FFA. 2024. Available at: https://www.iowaffa.com/about.aspx Accessed on 18 Jan 2024.

[CR43] Irwin A, Mihulkova J, Berkeley S, Tone L-R. “No-one else wears one:” exploring farmer attitudes towards all-Terrain vehicle helmets using the COM-B model. J Safety Res. 2022;81:123–33. 35589283 10.1016/j.jsr.2022.02.004

[CR44] Jennissen CA, Harland KK, Wetjen K, Peck J, Hoogerwerf P, Denning GM. A school-based study of adolescent all-terrain vehicle exposure, safety behaviors, and crash experience. Ann Fam Med. 2014;12(4):310–6. 25024238 10.1370/afm.1663 PMC4096467

[CR45] Jennissen CA, Harland KK, Wetjen K, Hoogerwerf P, O’Donnell L, Denning GM. All-terrain vehicle safety knowledge, riding behaviors and crash experience of Farm Progress Show attendees. J Safety Res. 2017;60:71–8. 28160816 10.1016/j.jsr.2016.12.001

[CR46] Jennissen CA, Denning GM, Aitken ME. A comprehensive report on all-terrain vehicles and youth: continuing challenges for injury prevention. Pediatrics. 2022;150(4):e2022059280. 36180617 10.1542/peds.2022-059280

[CR47] Jennissen CA, Champoux KL, Hoogerwerf PJ, Wetjen KM, Mulford LJ, Schaeffer SE, Okoro UE, Denning FM. All-terrain vehicle exposure and the association of ertified training on adolescent safety behaviors and crash experiences. Inj Epidemiol. 2022;9(Suppl 1):36. 36544186 10.1186/s40621-022-00404-7 PMC9768879

[CR48] Jinnah H, Stoneman Z. Age- and gender-based patterns in youth all-terrain vehicle (ATV) riding behaviors. J Agromed. 2016;21(2):163–70. 10.1080/1059924X.2016.1141736 26786819

[CR49] Keenan HT, Bratton SL. All-terrain vehicle legislation for children: a comparison of a state with and a state without a helmet law. Pediatrics. 2004;113(4):e330–4. 15060263 10.1542/peds.113.4.e330

[CR50] Keezer MR, Rughani A, Carroll M, Haas B. Head first: bicycle-helmet use and our children’s safety. Can Fam Physician. 2007;53(7):1131–2. 17872797 PMC1949278

[CR51] Kelleher CM, Metze SL, Dillon PA, Mychaliska GB, Keshen TH, Foglia RP. Unsafe at any speed–kids riding all-terrain vehicles. J Pediatr Surg. 2005;40(6):929–34. 15991173 10.1016/j.jpedsurg.2005.03.007

[CR52] Khambalia A, MacArthur C, Parkin PC. Peer and adult companion helmet use is associated with bicycle helmet use by children. Pediatrics. 2005;116(4):939–42. 16199705 10.1542/peds.2005-0518

[CR53] Khor D, Inaba K, Aiolfi A, Delapena S, Benjamin E, Matsushima K, Strumwasser AM, Demetriades D. The impact of helmet use on outcomes after a motorcycle crash. Injury. 2017;48(5):1093–7. 28242065 10.1016/j.injury.2017.02.006

[CR54] Kirkpatrick R, Puffinbarger W, Sullivan JA. All-terrain vehicle injuries in children. J Pediatr Orthop. 2007;27(7):725–8. 17878773 10.1097/BPO.0b013e3181558856

[CR55] Larson AN, McIntosh AL. The epidemiology of injury in ATV and motocross sports. Med Sport Sci. 2012;58:158–72. 22824845 10.1159/000338728

[CR56] Lawrence J, Kerns T, Burch C, Thomas A, Bell E. Motorcycle helmet use and head and facial injuries: crash outcomes in CODES-linked data. NHTSA. Oct 2009 (DOT HS 811 208). Available at: https://crashstats.nhtsa.dot.gov/Api/Public/ViewPublication/811208 Accessed 19 Jan 2024.

[CR57] Levy BE, Quattrone M, Castle JT, Doud AN, Draus JM, Worhunsky DJ. Injury pattern and outcomes following all-terrain vehicle accidents in Kentucky children: a retrospective study. Am Surg. 2023;89(12):5874–80. 37203181 10.1177/00031348231173955

[CR58] Linnaus ME, Ragar RL, Garvey EM, Fraser JD. Injuries and outcomes associated with recreational vehicle accidents in pediatric trauma. J Pediatr Surg. 2017;52(2):327–33. 27670961 10.1016/j.jpedsurg.2016.09.003

[CR59] Liu BC, Ivers R, Norton R, Boufous S, Blows S, Lo SK. Helmets for preventing injury in motorcycle riders. Cochrane Database Syst Rev. 2008;23(1):CD004333. 10.1002/14651858.CD004333.pub3 PMC13092890 18254047

[CR60] Mangano FT, Menendez JA, Smyth MD, Leonard JR, Narayan P, Park TS. Pediatric neurosurgical injuries associated with all-terrain vehicle accidents: a 10-year experience at St Louis Children’s Hospital. J Neurosurg. 2006;105(1 Suppl):2–5. 16871862 10.3171/ped.2006.105.1.2

[CR61] Mcintosh AS, Patton DA, Rechnitzer G, Grzebieta R. Injury mechanisms in fatal Australian quad bike incidents. Traffic Inj Prev. 2016;17(4):386–90. 26515914 10.1080/15389588.2015.1091073

[CR62] McIntosh AL, Christophersen CM. Motocross injuries in pediatric and adolescent patients. J Am Acad Orthop Surg. 2018;26(5):162–5. 29473832 10.5435/JAAOS-D-16-00405

[CR63] Merrigan TL, Wall PL, Smith HL, Janus TJ, Sidwell RA. The burden of unhelmeted and uninsured ATV drivers and passengers. Traffic Inj Prev. 2011;12(3):251–5. 21660891 10.1080/15389588.2011.561455

[CR64] Miller PA, Binns HJ, Christofffel KK. Children’s bicycle helmet attitudes and use. Association with parental rules. The Pediatric Practice Research Group. Arch Pediatr Adolesc Med. 1996;150(12):1259–64. 8953997 10.1001/archpedi.1996.02170370037005

[CR65] Miller B, Baig M, Hayes J, Elton S. Injury outcomes in children following automobile, motorcycle, and all-terrain vehicle accidents: an institutional review. J Neurosurg. 2006;105(3 Suppl):182–6. 16970230 10.3171/ped.2006.105.3.182

[CR66] Myers ML, Cole HP, Mazur JM. Cost effectiveness of wearing head protection on all-terrain vehicles. J Agromed. 2009;14(3):312–23. 10.1080/10599240903041885 19657881

[CR4] National Highway Traffic Safety Administration. Motorcycle safety 5-year plan. May 2019. (DOT HS 812 488). U.S. Department of Transportation, Washington, DC. Available at: https://www.nhtsa.gov/sites/nhtsa.gov/files/documents/13507-motorcycle_safety_plan_050919_v8-tag.pdf Accessed 2 Feb 2024.

[CR67] Nabaweesi R, Robbins JM, Goudie A, Onukwube JI, Bowman SM, Aitken ME. A cross-sectional study of emergency department visits by children after all-terrain vehicle crashes, motor vehicle crashes, and sports activities. Pediatr Emerg Care. 2018;34(7):479–83. 27383406 10.1097/PEC.0000000000000776

[CR68] National Highway Traffic Safety Administration (NHTSA). Traffic Safety Facts 2009 Data. December 2009. (DOT HS 811 392). U.S. Department of Transportation, Washington, DC. Available at: https://crashstats.nhtsa.dot.gov/Api/Public/ViewPublication/811392#:~:text=In%202009%2C%20there%20were%20an,crashes%20involved%20property%20damage%20only. Accessed 2 Feb 2024.

[CR69] National Highway Traffic Safety Administration (NHTSA). Motorcycles. Traffic Safety Facts. 2021. (DOT HS 813 112). U.S. Department of Transportation, Washington, DC. Available at: https://crashstats.nhtsa.dot.gov/Api/Public/ViewPublication/813112 Accessed 19 Jan 2024.

[CR70] Nichols JC, Sorrentino A, Hayslip M, King W, Jones A, Monroe K. Pediatric injury due to wheeled recreational devices: a single-institution retrospective study. Inj Epidemiol. 2022;9(Suppl 1):44. 36544196 10.1186/s40621-022-00395-5 PMC9768874

[CR71] Nortica DM, Sayrs LW, Krishna N, Davenport KP, Jamshidi R, McMahon L. Impact of helmet laws on motorcycle crash mortality rates. J Trauma Acute Care Surg. 2020;89(5):962–70. 33108139 10.1097/TA.0000000000002861

[CR72] Patel PB, Staley CA, Runner R, Mehta S, Schenker ML. Unhelmeted motorcycle riders have increased injury burden: a need to revisit universal helmet laws. J Surg Res. 2019;242:177–82. 31078903 10.1016/j.jss.2019.03.023

[CR73] Rodgers GB. The effectiveness of helmets in reducing all-terrain vehicle injuries and deaths. Accid Anal Prev. 1990;22(1):47–58. 2108691 10.1016/0001-4575(90)90006-7

[CR74] Shannon SF, Hernandez NM, Sems SA, Larson AN, Milbrandt TA. Pediatric orthopaedic trauma and associated injuries of snowmobile, ATV, and dirtbike accidents: a 19-year experience at a level 1 pediatric trauma center. J Pediatr Orthop. 2018;38(8):403–9. 27442216 10.1097/BPO.0000000000000838

[CR75] Shults RA, West BA. ATV riding and helmet use among youth aged 12–17 years, USA, 2011: results from the YouthStyles survey. Inj Prev. 2015;21(1):10–4. 24916683 10.1136/injuryprev-2013-041138

[CR76] Shults RA, Wiles SD, Vajani M, Helmkamp JC. All-terrain vehicle-related nonfatal injuries among young riders: United States, 2001–2003. Pediatrics. 2005;116(5):e608–12. 16263975 10.1542/peds.2005-0937

[CR77] Shults RA, West BA, Rudd RA, Helmkamp JC. All-terrain vehicle-related nonfatal injuries among young riders in the United States, 2001–2010. Pediatrics. 2013;132(2):282–9. 23821703 10.1542/peds.2013-0751 PMC5751408

[CR78] Testerman GM, Prior DC, Wells TD, Rollins SE, Oesch SL. Helmets matter: Kentucky all-terrain vehicle crashes seen at a Tennessee trauma center. Am Surg. 2018;84:289–93. 29580360 10.1177/000313481808400239

[CR79] Unni P, Morrow SE, Shultz L. Analysis of pediatric all-terrain vehicle trauma data in Middle Tennessee: implications for injury prevention. J Trauma Acute Care Surg. 2012;73(4 Suppl 3):S277–80. 23026968 10.1097/TA0b013e31826b00d7

[CR80] Vittetoe KL, Allen JH, Unni P, McKay KG, Yengo-Kahn AM, Ghani O, Mummidi P, Greeno AL, Bonfield CM, Lovvorn HN. Socioeconomic factors associated with helmet use in pediatric ATV and dirt bike trauma. Trauma Surg Acute Care Open. 2022;7(1):e000876. 35372699 10.1136/tsaco-2021-000876 PMC8928387

[CR81] Wiener RC, Waters C, Harper M, Trickett AK, Shockey RB. All-terrain vehicle-related emergency department visits: interaction of sex and age, NEISS, 2019. J Emerg Med. 2022;62(6):810–9. 10.1016/j.jemermed.2022.02.005. 35562243 10.1016/j.jemermed.2022.02.005 PMC9520756

[CR82] Wymore C, Denning G, Hoogerwerf P, Wetjen K, Jennissen C. Parental attitudes and family helmet use for all-terrain vehicles and bicycles. Inj Epidemiol. 2020;7(Suppl 1):23. 32532340 10.1186/s40621-020-00253-2 PMC7291627

[CR84] Zhang C. Report of deaths and injuries involving off-highway vehicles with more than two vehicles. United States Consumer Product Safety Commission. May 2023. 2022. Available at: https://www.cpsc.gov/s3fs-public/OHV-Annual-Report-2022.pdf Accessed on 17 Jan 2024.

